# Supplementary material for: The vibriophage-encoded inhibitor OrbA abrogates BREX-mediated defense through the ATPase BrxC
Source: J Bacteriol. 2024 Oct 15;206(11):e00206-24. doi: 10.1128/jb.00206-24 (PMC11580459; doi:10.1128/jb.00206-24)
Supplement: Supplemental tables and figures — Tables S2 to S3; Fig. S1 to S7. [file jb.00206-24-s0001.pdf]

Table S2: Strains

Strain	Genotype[a]	Reference
KDS6	V. cholerae E7946, SXT(-), Sm^R, Tm^S (O1, El Tor biotype) (referred to as permissive in text)	Laboratory collection
KL560	V. cholerae E7946 Δ*lacZ::kan*, ICEVchInd5 ΔHS5::frt, Sm^R, Tm^R, Kan^R (referred to as ΔBREX in text)	[1]
KS1876	V. cholerae E7946, SXT(-) pKL06:empty vector Sm^R, Cm^R, Tm^S	[1]
KS2461	V. cholerae E7946 Δ*lacZ::kan*, ICEVchInd5 ΔfloR::frt, Sm^R, Tm^R, Kan^R (referred to as BREX in text)	[1]
KS2471	V. cholerae E7946 Δ*lacZ::kan*, ICEVchInd5 ΔfloR::frt, pGp25 (vector is pKL06::*orbA*), Sm^R, Tm^R, Kan^R,Cm^R	[1]
KS2490	V. cholerae E7946 Δ*lacZ::kan*, ICEVchInd5 ΔfloR::frt, pKL06:empty vector; Sm^R, Tm^R, Kan^R,Cm^R	[1]
RO24	V. cholerae E7946 Δ*lacZ::kan*, ICEVchInd5 ΔfloR::frt pRO01 Sm^R, Tm^R, Kan^R,Cm^R	This study
RO25	V. cholerae E7946 Δ*lacZ::kan*, ICEVchInd5 ΔfloR::frt pRO02 cm, SmR, TmR, KanR	This study
RO31	V. cholerae E7946 Δ*lacZ::kan*, ICEVchInd5 ΔfloR::frt pCMB17 Sm^R, Tm^R, Kan^R,Cm^R	This study
RO32	V. cholerae E7946 Δ*lacZ::kan*, ICEVchInd5 ΔfloR (clean deletion, no frt scar) SmR, TmR, KanR	This study
RO38	*V. cholerae* E7949 Δ*lacZ::kan*, ICE*Vch*Ban9 ΔfloR Sm^R, Tm^R, Kan^R,	This study
RO46-47	V. cholerae E7946 Δ*lacZ::kan*, ICEVchInd5 Δ*brxC*::*frt-spec* ΔfloR Sm^R, Tm^R, Kan^R,$Spec^R$	This study
RO50-51	V. cholerae E7946 Δ*lacZ::kan*, ICEVchInd5 Δ*brxC*::*frt* ΔfloR Sm^R, Tm^R, Kan^R	This study
RO53	V. cholerae E7946 ΔlacZ::Kan, ICEVchInd5 Δ*brxC*::*frt* ΔfloR pRO11 Sm^R, Tm^R, Kan^R, Cm^R	This study
RO54	V. cholerae E7946 Δ*lacZ::kan*, ICEVchInd5 Δ*brxC*::*frt* ΔfloR pRO24 Sm^R, Tm^R, Kan^R, Cm^R	This study
RO56	V. cholerae E7946 Δ*lacZ::kan*, ICEVchInd5 Δ*brxC*::*frt* ΔfloR pKL06:empty vector cm, SmR, TmR, KanR	This study
RO152	V. cholerae E7946 Δ*lacZ::kan*, ICEVchInd5 Δ*brxC*::*frt* ΔfloR pRO22 Sm^R, Tm^R, Kan^R, Cm^R	This study
RO153	V. cholerae E7946 Δ*lacZ::kan*, ICEVchInd5 ΔfloR pRO24 Sm^R, Tm^R, Kan^R, Cm^R	This study
RO177	V. cholerae E7946 Δ*lacZ::kan*, ICEVchInd5 Δ*brxC*::*frt* ΔfloR pCMB17 Sm^R, Tm^R, Kan^R, Cm^R	This study

RO234	V. cholerae E7946 Δ*lacZ::kan*, ICEVchInd5 Δ*brxC*::*frt* ΔfloR pRO76 *Sm*^{R}, *Tm*^{R}, *Kan*^{R}, Cm^{R}	This study
RO235	V. cholerae E7946 Δ*lacZ::kan*, ICEVchInd5 Δ*brxC*::*frt* ΔfloR pRO62 *Sm*^{R}, *Tm*^{R}, *Kan*^{R}, Cm^{R}	This study
RO236	V. cholerae E7946 Δ*lacZ::kan*, ICEVchInd5 Δ*brxC*::*frt* ΔfloR pRO77 *Sm*^{R}, *Tm*^{R}, *Kan*^{R}, Cm^{R}	This study
RO243	V. cholerae E7946 Δ*lacZ::kan*, ICEVchInd5 ΔfloR pRO62 SmR, TmR, KanR	This study
RO250	V. cholerae E7946 ΔlacZ::Kan, ICEVchInd5 ΔfloR pRO11 *Sm*^{R}, *Tm*^{R}, *Kan*^{R}, Cm^{R}	This study
RO251	V. cholerae E7946 Δ*lacZ::kan*, ICEVchInd5 ΔfloR pKL06 *Sm*^{R}, *Tm*^{R}, *Kan*^{R}, Cm^{R}	This study
RO335-336	*V. cholerae* E7946 Δ*lacZ::kan*, ICE*Vch*Ban9 Δ*brxU*::*frt-spec* ΔfloR *Sm*^{R}, *Tm*^{R}, *Kan*^{R}, *Spec*^{R}	This study
RO389	*V. cholerae* E7946 Δ*lacZ::kan*, ICEVchInd5 Δ*brxC*::*frt* ΔfloR pRO37 *Sm*^{R}, *Tm*^{R}, *Kan*^{R}, Cm^{R}	This study
RO390-391	*V. cholerae* E7949 Δ*lacZ::kan*, ICE*Vch*Ban9 Δ*brxC*::*frt-spec* ΔfloR *Sm*^{R}, *Tm*^{R}, *Kan*^{R}, *Spec*^{R}	This study
RO394	*V. cholerae* E7949 Δ*lacZ::kan*, ICE*Vch*Ban9 Δ*brxC*::*frt* ΔfloR *Sm*^{R}, *Tm*^{R}, *Kan*^{R}	This study
RO395	*V. cholerae* E7949 Δ*lacZ::kan*, ICE*Vch*Ban9 Δ*brxC*::*frt* ΔfloR pRO11 *Sm*^{R}, *Tm*^{R}, *Kan*^{R}, *Cm*^{R}	This study
RO396	*V. cholerae* E7949 Δ*lacZ::kan*, ICE*Vch*Ban9 Δ*brxC*::*frt* ΔfloR pRO37 *Sm*^{R}, *Tm*^{R}, *Kan*^{R}, *Cm*^{R}	This study
RO397	*V. cholerae* E7949 Δ*lacZ::kan*, ICE*Vch*Ban9 Δ*brxC*::*frt* ΔfloR pKL06 *Sm*^{R}, *Tm*^{R}, *Kan*^{R}, *Cm*^{R}	This study
RO554	*V. cholerae* E7946 Δ*lacZ::kan*, ICE*Vch*Ban9 Δ*brxU*::*frt* ΔfloR *Sm*^{R}, *Tm*^{R}, *Kan*^{R}	This study
Phage	Description	Reference
ICP1_2006_E	ICP1 phage isolate (accession number MH310934; referred to as ICP1)	[2]
ICP1_2006_E Δ*orbA* (ROΦ1)	ICP1_2006_E with a deletion in the *orbA* gene, referred to ICP1 Δ*orbA*	[1]

[a] Streptomycin – *Sm*; Kanamycin – *Kan*; Spectinomycin – *Spec*; Chloramphenicol – *Cm*; Trimethoprim – *Tm*; Resistance – R; Sensitive – S

Table S3: Plasmids

Plasmids	*E. coli* Strain	Genotype^{a}	Reference
pCMB17	CMB17 (*E. coli* S17)	Ptac-riboE-3xFLAG (codon optimized), *Cm*^{R}	This study
pFlippase	SGH109 (*E. coli* S17)	Ptac-riboE-flippase, *Cm*^{R}	[1]
pGp25(*orbA*)	KS2432 (*E. coli* S17)	Ptac-riboE-gp25 (*orbA*; (ADX87841.1)), *Cm*^{R}	[1]
pKL06	KS1870 (*E. coli* S17)	Ptac-riboE-empty vector, *Cm*^{R}	[1]
pRO01	RO87 (*E. coli* S17)	Ptac-riboE-*orbA-3xFLAG, Cm*^{R}	This study
pRO02	RO88 (*E. coli* S17)	Ptac-riboE-3xFLAG-*orbA, Cm*^{R}	This study
pRO11	RO106 (*E. coli* S17)	Ptac-riboE-*brxC*(*Vch*Ind5)*, Cm*^{R}	This study
pRO22	RO151 (*E. coli* S17)	Ptac-riboE-*brxC-3xFLAG* (*Vch*Ind5)*, Cm*^{R}	This study
pRO24	RO118 (*E. coli* S17)	Ptac-riboE-*brxC(K73A)* (*Vch*Ind5)*, Cm*^{R}	This study
pRO37	RO383 (*E. coli* S17)	Ptac-riboE-*brxC* (*Vch*Ban9)*, Cm*^{R}	This study
pRO62	RO231 (*E. coli* S17)	Ptac-riboE-*brxC*^{E255A} (*Vch*Ind5)*, Cm*^{R}	This study
pRO76	RO229 (*E. coli* S17)	Ptac-riboE-*brxC*^{E255A}*-3xFLAG* (*Vch*Ind5)*, Cm*^{R}	This study
pRO77	RO233 (*E. coli* S17)	Ptac-riboE-*brxC*^{K73A}*-3xFLAG* (*Vch*Ind5)*, Cm*^{R}	This study
pRO97	RO434 (*E. coli* XL1-Blue)	T-18-N-linker-*brxC* (*Vch*Ind5), *Amp*^{R}	This study
pRO98	RO435 (*E. coli* XL1-Blue)	T-18C-linker-*brxC* (*Vch*Ind5), *Amp*^{R}	This study
pRO99	RO436 (*E. coli* XL1-Blue)	T-25-N-linker-*brxC* (*Vch*Ind5), *Kan*^{R}	This study
pRO100	RO437 (*E. coli* XL1-Blue)	T-25-C-linker-*brxC* (*Vch*Ind5), *Kan*^{R}	This study
pRO101	RO414 (*E. coli* XL1-Blue)	T-18-N-linker-*orbA*, *Amp*^{R}	This study
pRO102	RO415 (*E. coli* XL1-Blue)	T-18-C-linker-*orbA*, *Amp*^{R}	This study
pRO103	RO416 (*E. coli* XL1-Blue)	T-25-N-linker-*orbA*, *Kan*^{R}	This study
pRO104	RO417 (*E. coli* XL1-Blue)	T-25-C-linker-*orbA*, *Kan*^{R}	This study
pRO119	RO455 (*E. coli* XL1-Blue)	T-18-N-linker-*brxC*^{K73A}(*Vch*Ind5), *Amp*^{R}	This study
pRO120	RO457 (*E. coli* XL1-Blue)	T-18-C-linker-*brxC*^{K73A} (*Vch*Ind5), *Amp*^{R}	This study
pRO121	RO458 (*E. coli* XL1-Blue)	T-25-N-linker-*brxC*^{K73A} (*Vch*Ind5), *Kan*^{R}	This study
pRO122	RO467 (*E. coli* XL1-Blue)	T-25-C-linker-*brxC*^{K73A} (*Vch*Ind5), *Kan*^{R}	This study
pRO123	RO460 (*E. coli* XL1-Blue)	T-18-N-linker-*brxC*^{E255A}(*Vch*Ind5), *Amp*^{R}	This study

pRO124	RO462 (*E. coli* XL1-Blue)	T-18-C-linker-*brxC*^{E255A} (*Vch*Ind5), *Amp*^{R}	This study
pRO125	RO463 (*E. coli* XL1-Blue)	T-25-N-linker-*brxC*^{E255A} (*Vch*Ind5), *Kan*^{R}	This study
pRO126	RO465 (*E. coli* XL1-Blue)	T-25-C-linker-*brxC*^{E255A} (*Vch*Ind5), *Kan*^{R}	This study
pRO127	RO507 (*E. coli* XL1-Blue)	T-18-N-linker-*brxC* (*Vch*Ban9), *Amp*^{R}	This study
pRO128	RO514 (*E. coli* XL1-Blue)	T-18-C-linker-*brxC* (*Vch*Ban9), *Amp*^{R}	This study
pRO129	RO508 (*E. coli* XL1-Blue)	T-25-N-linker-*brxC* (*Vch*Ban9), *Kan*^{R}	This study
pRO130	RO510 (*E. coli* XL1-Blue)	T-25-C-linker-*brxC* (*Vch*Ban9), Kan^{R}	This study
pRO158	RO518 (*E. coli* XL1-Blue)	T-18-N-linker-*brxC* (*Vch*Ind5) + *rbs-orbA*, *Amp*^{R}	This study
pRO159	RO520 (*E. coli* XL1-Blue)	T-18-C-linker-*brxC* (*Vch*Ind5) + *rbs-orbA*, *Amp*^{R}	This study

[a] Kanamycin – *Kan*; Chloramphenicol – *Cm*; Ampicillin – *Amp*; Resistance – R

Supplemental References:

1. LeGault, K. N. *et al.* Temporal shifts in antibiotic resistance elements govern phage-pathogen conflicts. *Science* **373**, eabg2166 (2021).

2. Seed, K. D., Lazinski, D. W., Calderwood, S. B. & Camilli, A. A bacteriophage encodes its own CRISPR/Cas adaptive response to evade host innate immunity. *Nature* **494**, 489–491 (2013).

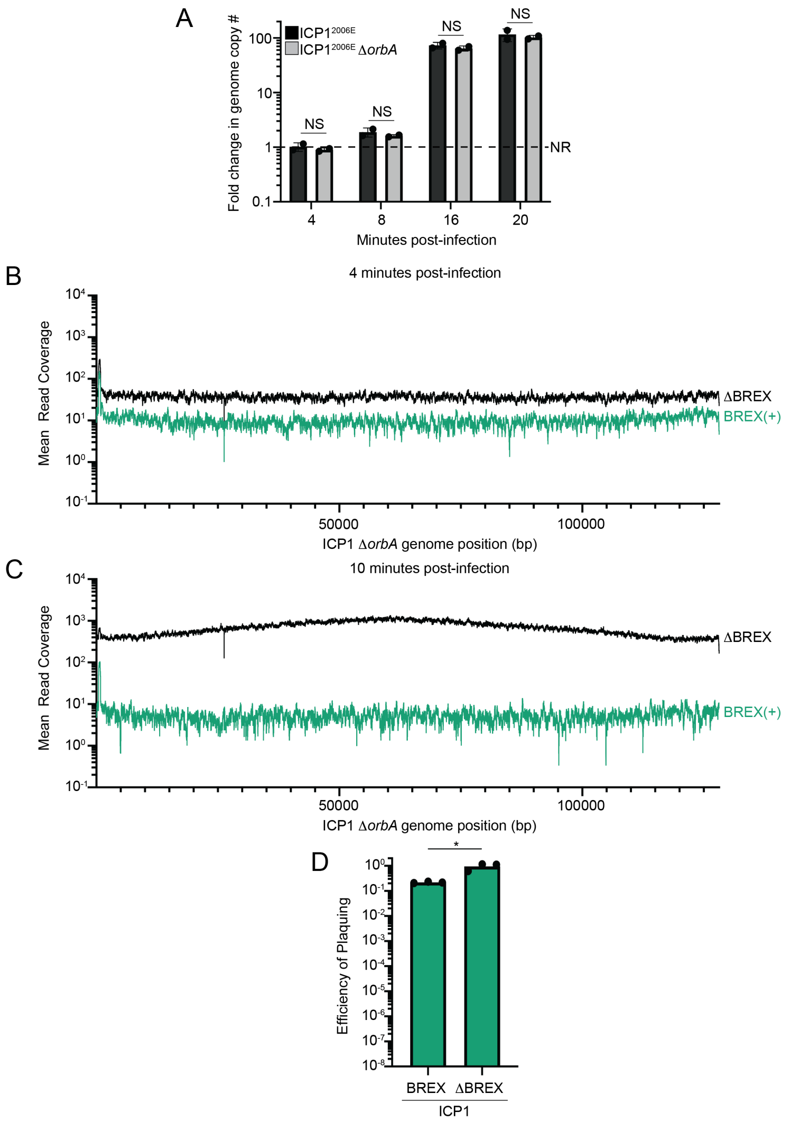

Supplemental Figure 1. BREX restricts phage genome replication while OrbA restores phage plaquing. A) Quantification of ICP1 and ICP1 Δ*orbA* replication in a permissive background. NR - no replication. Statistical analysis was performed using a student t-test (* = p<0.01, ** = p<0.001, *** = p<0.0001, **** p=<0.00001). NS – not significant. **B-C)** Mean read coverage across the ICP1 Δ*orbA* genome in the presence and absence of BREX at **B)** 4 minutes post-infection and **C)** 10 minutes post-infection. *V. cholerae* cells either harboring the SXT *Vch*Ind5 (BREX(+); green) or where the BREX system and all of hotspot 5 were deleted (ΔBREX; black) were infected with ICP1 Δ*orbA*. The coverage shown is the mean of three biological replicates. **D)** Efficiency of plaquing (EOP) of ICP1 on *V. cholerae* that encodes the *Vch*Ind5 BREX system and deleted for the BREX system relative to the permissive background E7946 lacking *Vch*Ind5. Each dot represents an individual biological replicate.

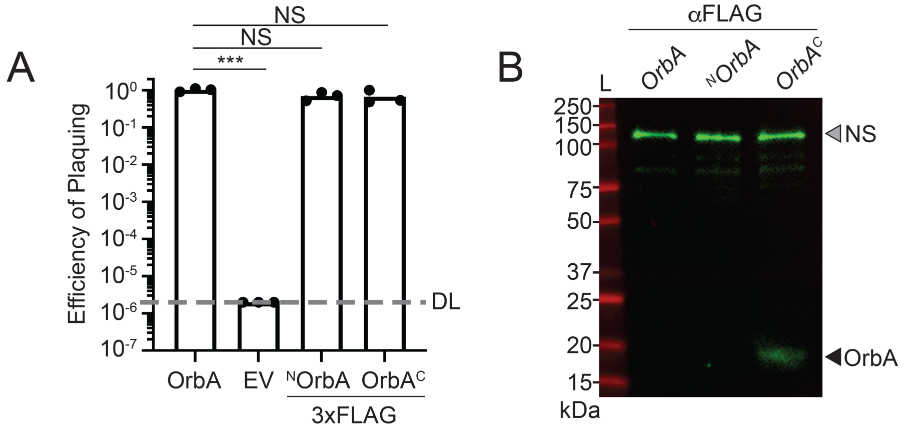

Supplemental Figure 2. FLAG-tagged OrbA can inhibit BREX. A) Efficiency of plaquing (EOP) of ICP1 Δ*orbA* on *V. cholerae* that encodes the *Vch*Ind5 BREX system carrying the vector indicated for expression of *orbA in trans* relative to the permissive strain *V. cholerae* E7946 lacking *Vch*Ind5 and harboring an empty vector. DL - detection limit. EV - empty vector. Untagged OrbA (OrbA). 3xFLAG translationally fused to the N-terminus of OrbA (^{N}OrbA). 3xFLAG translationally fused to the C-terminus of OrbA (OrbA^{C}) Each dot represents a biological replicate. Statistical analysis was performed using a one-way ANOVA (* = p<0.01, ** = p<0.001, *** = p<0.0001, **** p=<0.00001). **B)** Western blot analysis of FLAG-tagged OrbA using a primary antibody that recognizes the 3xFLAG tag. The contaminating non-specific protein (NS) recognized by the primary anti-FLAG antibody is used as a loading control. The ladder (L) indicates the estimated molecular weight of the visualized proteins.

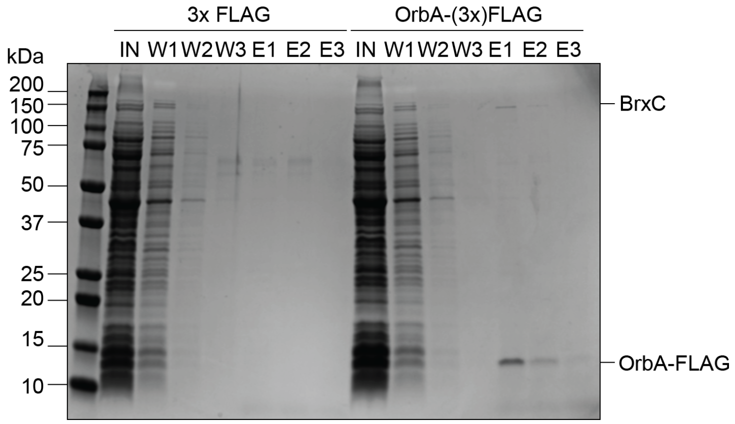

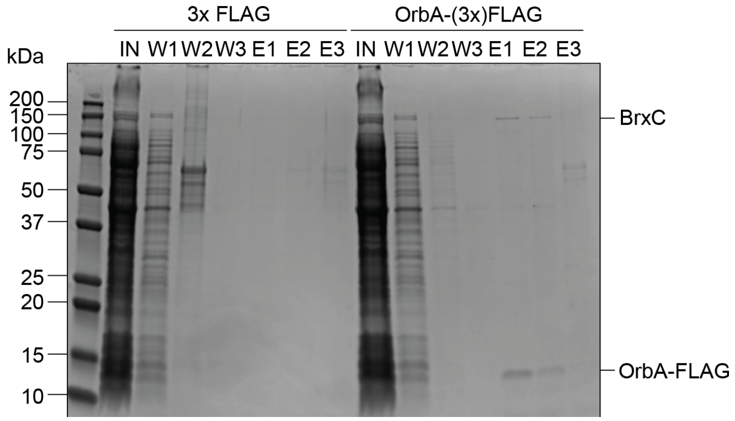

Supplemental Figure 3. Gel images of samples from replicates of 3xFLAG and OrbA-3xFLAG CoIPs resolved by SDS-PAGE and stained with Coomassie brilliant blue. IN – input. W – Wash. E – Elutions.

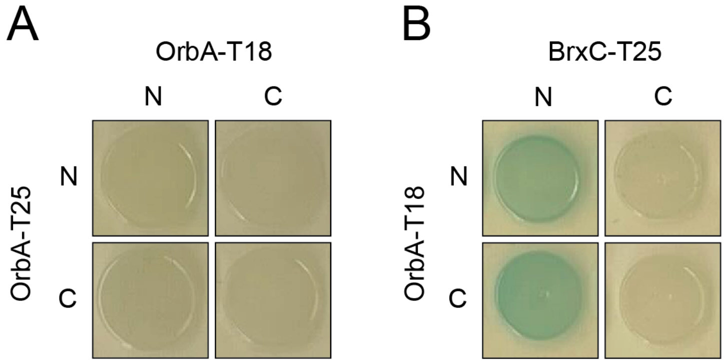

Supplemental Figure 4. OrbA interacts with BrxC. **A and B)** Bacterial two-hybrid analysis of the interaction between **A)** OrbA-OrbA and **B)** BrxC-OrbA. N- and C- indicate the terminus of each half of the adenylate cyclase enzyme was translationally fused to the protein of interest.

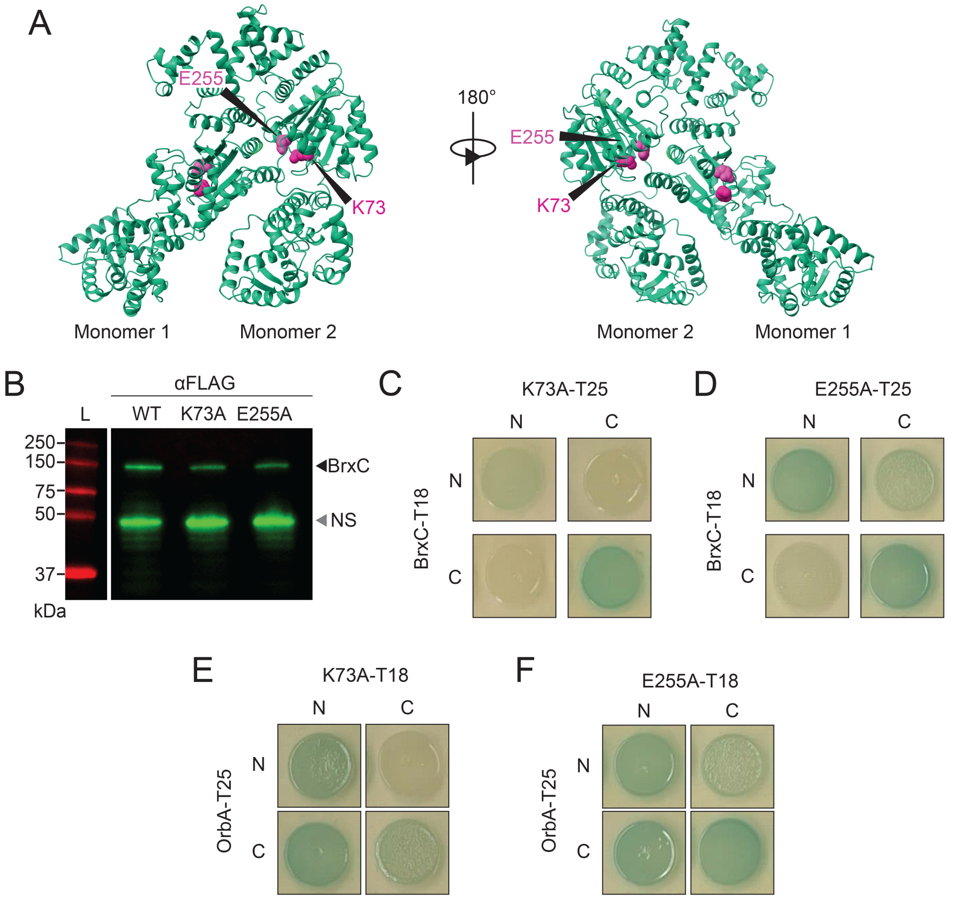

Supplemental Figure 5. Mutation of the Walker A and B motif alters BrxC interactions. A) Structural prediction of the N-terminal region of BrxC (1-532) as a dimer using ColabFold. Each BrxC monomer is indicated. The residues that were mutated in the Walker A and B motifs, K73 (dark pink) and E255 (light pink), respectively, are labeled on each monomer. **B)** Western blot analysis of either wild-type BrxC translationally fused to a C-terminal 3xFLAG tag or the Walker A (K73A) or Walker B (E255A) mutants using a primary antibody against the FLAG tag. NS - non-specific *V. cholerae* contaminating band used as a loading control. The ladder (L) indicates the estimated molecular weight of the visualized proteins. **C-F)** Bacterial two-hybrid analysis of the interaction between **C)** $BrxC^{WT}$-$BrxC^{K73A}$, **D)** $BrxC^{WT}$-$BrxC^{E255A}$, **E)** $BrxC^{K73A}$-OrbA, and **F)** $BrxC^{E255A}$-OrbA. N- and C- indicate the terminus of each half of the adenylate cyclase enzyme was fused to the protein of interest.

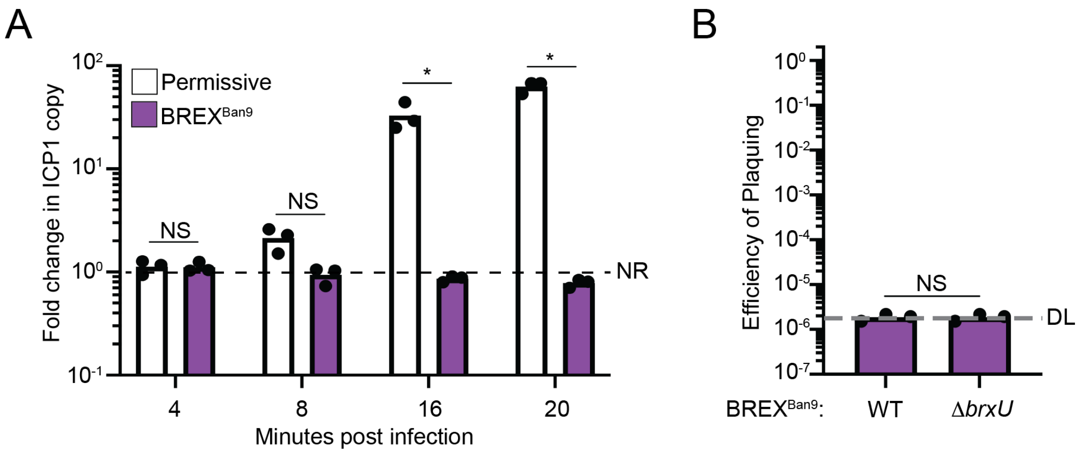

Supplemental Figure 6. BrxU is not required for the inhibition of ICP1 by BREX^{Ban9} A) Quantification of ICP1 genome replication in the presence and absence of BREX^{Ban9}. Samples taken immediately following the addition of phage (Time 0) were compared to the indicated time points to calculate fold change. NR - no replication. Statistical analysis was performed using a student t-test was performed (* = p<0.01, ** = p<0.001, *** = p<0.0001, **** p=<0.00001). NS – not significant. Each dot represents a biological replicate **B)** Efficiency of plaquing (EOP) of ICP1 on *V. cholerae* either encoding a wild-type BREX system from *Vch*Ban9 (BREX^{Ban9}) or a BREX system from *Vch*Ban9 with *brxU* deleted (Δ*brxU*) relative to the permissive strain E7946 lacking *Vch*Ban9. DL - detection limit. Statistical analysis was performed using a student t-test (* = p<0.01, ** = p<0.001, *** = p<0.0001, **** p=<0.00001). NS – not significant. Each dot represents an individual biological replicate.

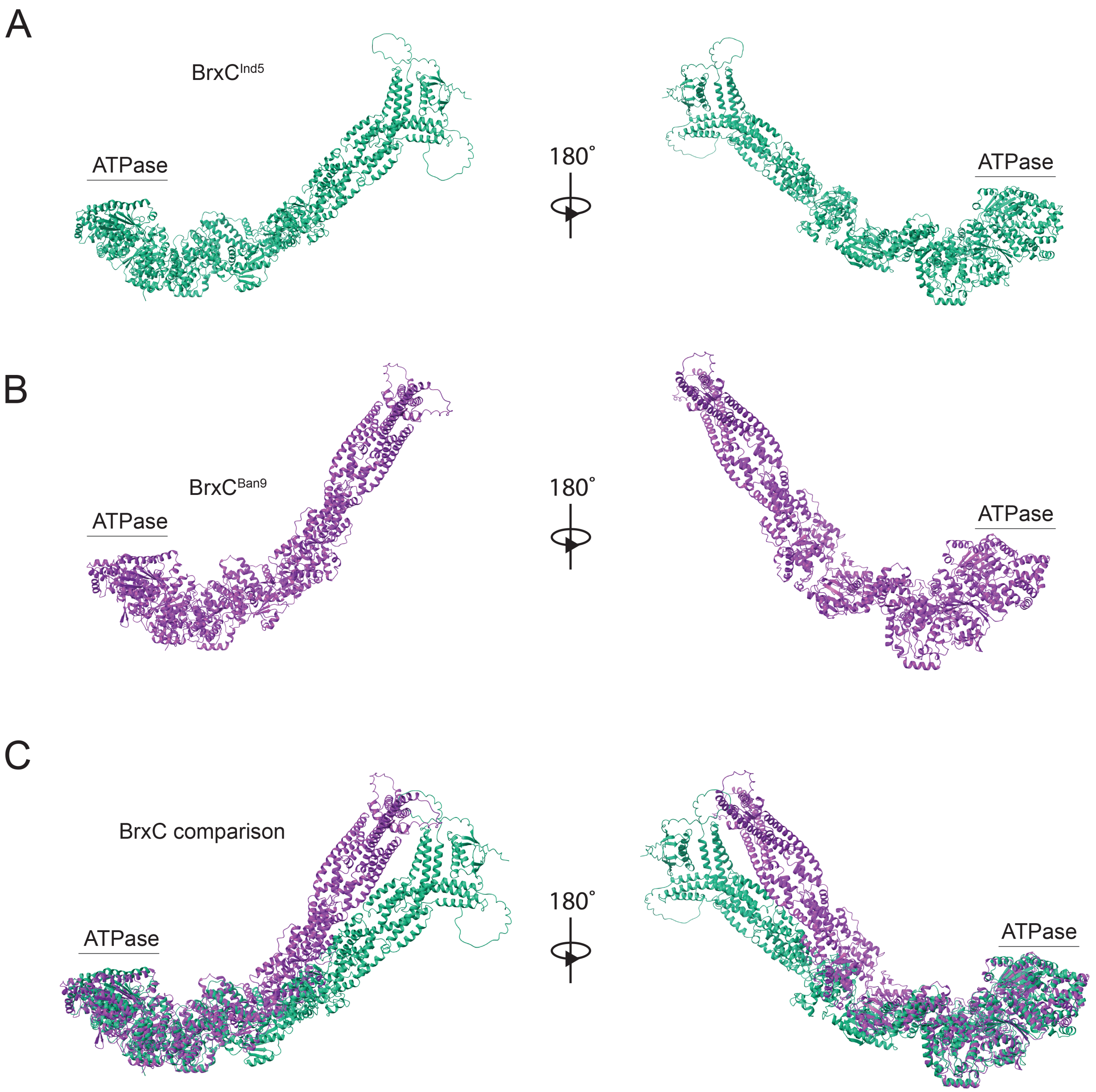

Supplemental Figure 7. The predicted structure of BrxC^{Ind5} is similar to BrxC^{Ban9}. A and B) Predicted structures of BrxC encoded by **A)** *Vch*Ind5 (BrxC^{Ind5}) and **B)** *Vch*Ban9 (BrxC^{Ban9}) determined by ColabFold **C)** Structural comparison of the two BrxC proteins by Chimera. The N-terminal ATPase domain of both proteins is indicated.
